# Supplementary figures and images for: A curated data resource of 214K metagenomes for characterization of the global antimicrobial resistome
Source: PLoS Biol. 2022 Sep 6;20(9):e3001792. doi: 10.1371/journal.pbio.3001792 (PMC9447899; doi:10.1371/journal.pbio.3001792)

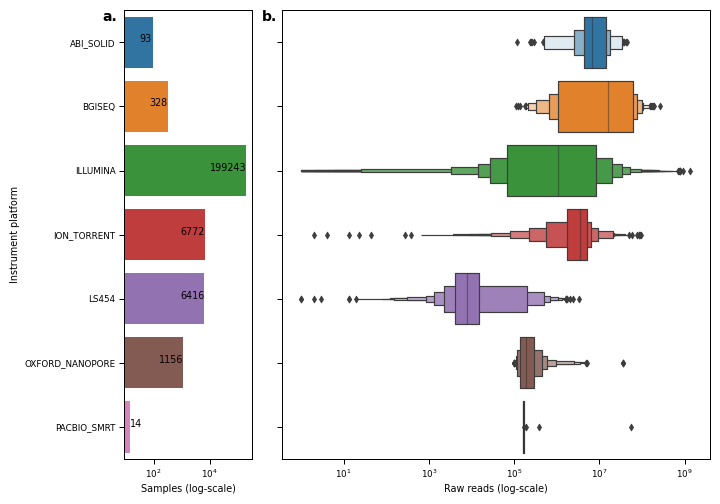

Supplement: S1 Fig — (a) Sample count per platform. (b) Distribution of raw sequencing read counts per platform. The data underlying this figure can be found at https://doi.org/10.5281/zenodo.6919377. (TIFF) [file pbio.3001792.s001.tiff]

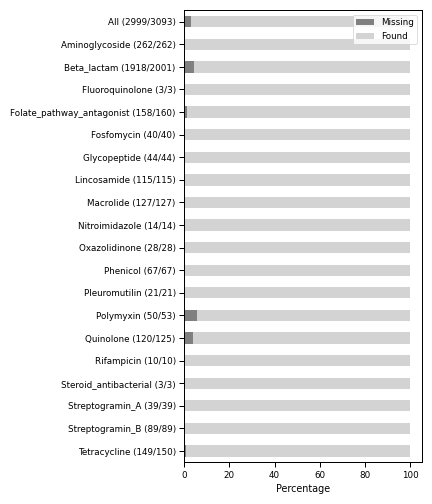

Supplement: S2 Fig — The bars illustrate the percentage of ARGs per resistance class without and with at least 1 aligned fragment. The parenthesis after each class label contains the number of genes found out of the total available templates. The data underlying this figure can be found at https://doi.org/10.5281/zenodo.6919377. (TIFF) [file pbio.3001792.s002.tiff]

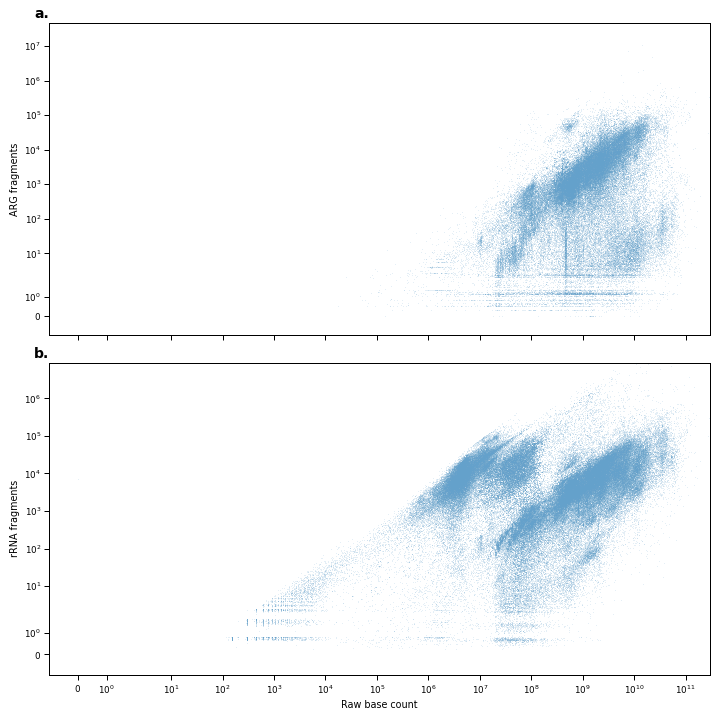

Supplement: S3 Fig — The sample-wise distribution of aligned (a) ARG or (b) rRNA fragments compared to raw sequencing base counts. The data underlying this figure can be found at https://doi.org/10.5281/zenodo.6919377. (TIFF) [file pbio.3001792.s003.tiff]

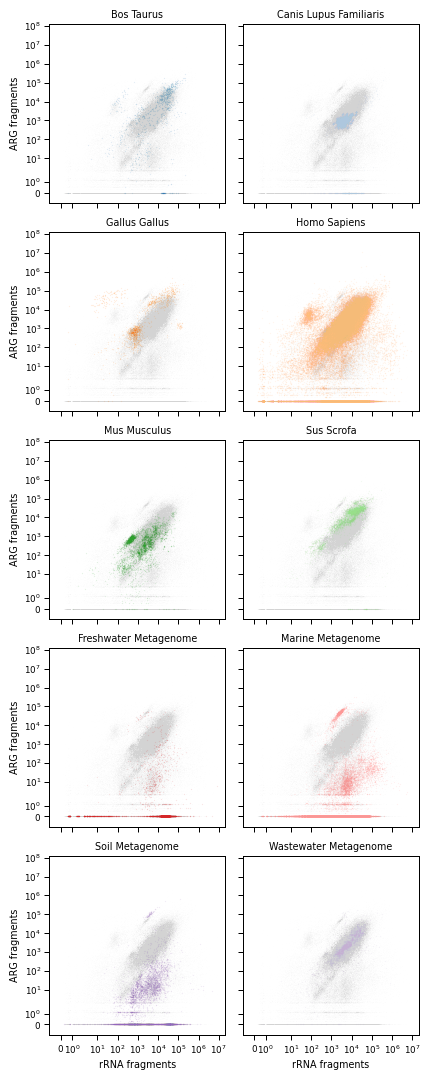

Supplement: S4 Fig — The data underlying this figure can be found at https://doi.org/10.5281/zenodo.6919377. (TIFF) [file pbio.3001792.s004.tiff]

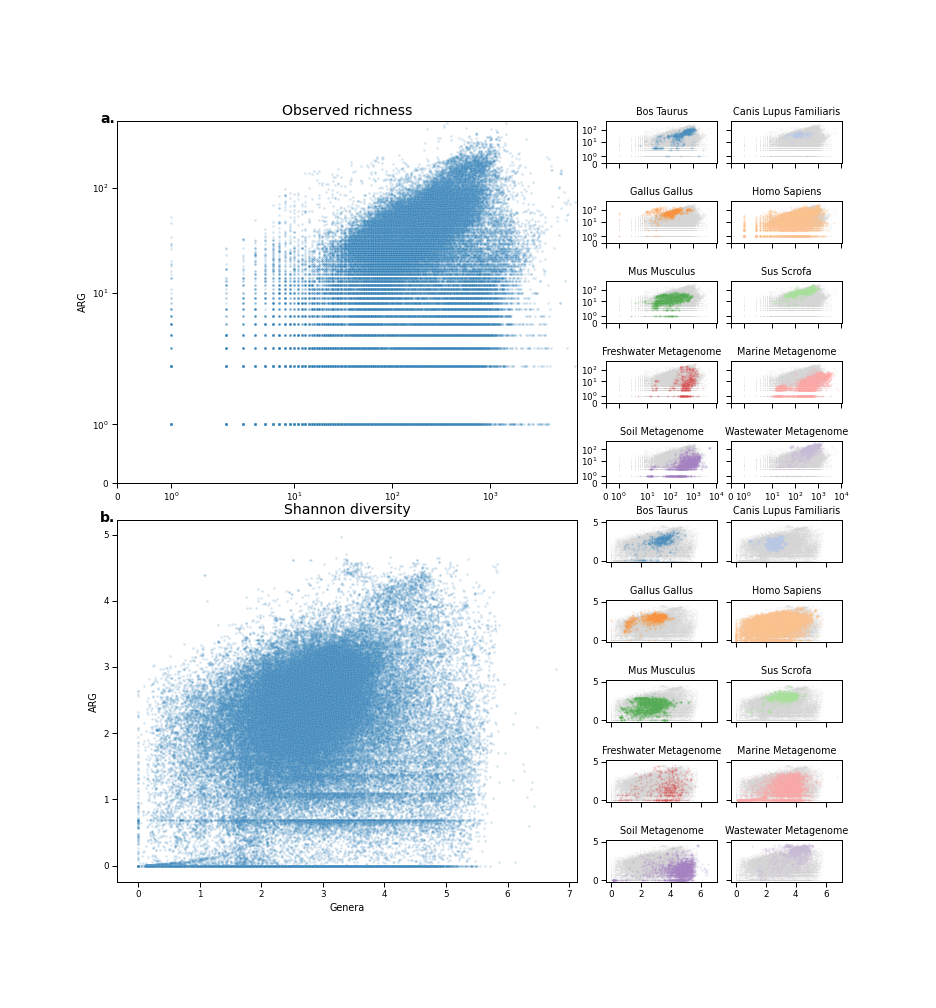

Supplement: S5 Fig — (a) The richness of genus groups (x-axis) vs. ARG richness (y-axis). (b) The relationship between Shannon diversity index calculated on genus level (x-axis) and ARGs (y-axis). Right: samples colored by selected host or environmental origins. The data underlying this figure can be found at https://doi.org/10.5281/zenodo.6919377. (TIFF) [file pbio.3001792.s005.tiff]
