# Supplementary material for: A curated data resource of 214K metagenomes for characterization of the global antimicrobial resistome
Source: PLoS Biol. 2022 Sep 6;20(9):e3001792. doi: 10.1371/journal.pbio.3001792 (PMC9447899; doi:10.1371/journal.pbio.3001792)
Supplement: Attachment — Submitted filename: response_PLOS_Biology.pdf [file pbio.3001792.s006.pdf]

We greatly appreciate the time the three reviewers have taken to comment on our manuscript, and we have incorporated several of their suggestions in our revised submission. The changes in the manuscript related to reviewer 1 are highlighted in red, reviewer 2 in blue, and reviewer 3 in green. Changes related to the editor's request or others are highlighted in orange. Below, you will find our point-by-point response to the reviewers' comments in *italics*.

Since a lot of the comments were either about clarifying our methods and expanding our discussion with excellent topics, the updated manuscript contains a longer discussion than the first version. Finally, we have clarified parts of the results sections as per requests of the reviewers.

Reply to editor's requests

b) Please could you change your Title to something slightly more explicit for our wider readership? We suggest "A curated data resource of 214K metagenomes characterizes the global antimicrobial resistome".

The title has been changed to "A curated data resource of 214K metagenomes for characterization of the global antimicrobial resistome", as while we give a general overview of AMR trends in the data, there are multiple other directions one can investigate, so we propose to keep the "for characterization of", instead of using "characterizes".

c) Please address my Data Policy requests below; specifically, we need you to supply the numerical values underlying Figs 1ABC, 2AB, 3ABCD, 4ABC, 5, S1AB, S2, S3, S4AB. I note that your Zenodo deposition currently only seems to contain relatively "raw" values, rather than those directly shown in the Figure – please could you include the latter, clearly labelled? If you've used any custom code, please also include this.
We have uploaded the numerical values used in the various figures to Zenodo and clearly labeled which file refers to which figure in the ZIP file named "figure_data.zip"– see https://zenodo.org/record/6919377/preview/figure_data.zip#tree_item0.

d) Please also cite the location of the data clearly in each main and supplementary Fig legend, e.g. "The data underlying this Figure can be found in https://doi.org/10.5281/zenodo.6519844"
We have accommodated this request by adding the Zenodo DOI in each figure legend.

Reply to reviewer 1

Martiny et al. describe a new data resource that is the product of intensive and large-scale bioinformatics analysis of metagenomic data for the presence and abundance of acquired antimicrobial resistance genes (ARGs). This paper can be viewed from two perspectives: (1) a data science contribution to allow the community to better examine AMR transmission patterns and (2) knowledge gained from analysis of the data.

DATA SCIENCE

From the data science perspective, this is a very significant contribution to the field using the latest standards and mining of >200,000 metagenomic datasets, totalling more than 400 TB of sequence data. Considerable effort was put into data harmonization and normalization to provide a high-value data set to AMR researchers. The data, pipelines, software, and results are provided in a well-organized open format, allowing their analysis by the broader community. As the amount of computation needed to produce this data set is well beyond most AMR researchers interested in using genomics to understand ARG transmission patterns, this contribution is novel and of high value.

Software and their versions are properly described in the methods section, but parameters used for KMA are not outlined. Were default parameters used? It is fine that the methods are presented "in brief" given a citation of previous work, but the manuscript would be improved if this included the cut-offs used by KMA to determine aligned reads (MAPQ?). Similarly, a more explicit statement in the methods that use of ResFinder focuses on the analysis of acquired ARGs and does not include resistance via mutation (e.g. PointFinder) would be helpful.

We have added the parameters used in the KMA procedure (lines 95-98) and have also defined that the ResFinder database consists of acquired ARGs (line 93).

ANALYSIS

Analysis and interpretation of the data are thin, with some issues that need to be addressed, but this does not undermine that the primary purpose of the manuscript is to describe the generation and content of the data produced for the broader community. Full analyses of these data are beyond the scope of this manuscript and the authors perform an adequate overview analysis and summary of the major sub-sets and trends in the data. However, the statement of "a general trend" in lines 227-229 does not appear supported by Figures 5 & S4. This section should be re-written to carefully discuss patterns supported by the data, such as exists for the chicken data, instead of broad statements based on unconvincing patterns in the plots.
We agreed that our wording was relatively poor and have therefore changed it to better highlight that several of the samples did both have high microbial and ARG diversity, but that is not true for all of them. The changes can be found in lines 234-239.

The data include ARGs that have as little as a single read fragment aligned and these ARGs were used in the species richness estimates. Can the authors explain why they did not include a minimum coverage cut-off in these analyses?

It is a good point but deciding what value to use as a minimum coverage will most likely change depending on the aim. For the sake of transparency in this paper, we did not do any filtering on the coverage. Instead, we decided to include the number of positions covered in the reference sequence in the data tables shared on Zenodo so that other potential users can decide on their filters. We have included this in the discussion in lines 284-287.

The results presented are broken down by host (i.e. environment), location, and ResFinder drug class, but not by ARG families. While others are very likely to analyze these data for

transmission patterns of ARGs or ARG families, at least an anecdotal investigation of a few ARGs would help illustrate the value of the data. Perhaps something recent like MCR versus the AACs? The "trends" mentioned above may be more obvious at the level ARG families.

Since the point of this paper is to introduce the trends in this data and give the reader ideas on what they can investigate, we have only given a brief overview of the trends broken down by metadata groups. However, we have previously published an in-depth study on the distribution of mcr genes in this data collection (Martiny 2022; 10.1128/msystems.00105-22), which also showcases how this resource can be used for specific gene families. We have added this study as an example of how the resource can be used in the discussion, see lines 264-267.

DISCUSSION

Successful annotation of metagenomics data for ARGS requires both good software for sequencing read alignment and good reference data. Both KMA and ResFinder reflect the latest standards but like CARD and other databases, ResFinder's reference data is primarily from clinical isolates. It is possible there are ARGs in the environmental metagenomics data that are sufficiently different from these reference data to a degree that KMA is unsuccessful. CARD has its "Resistomes & Variants" data to provide an alternate in silico diversity of >200,000 ARG alleles for sequence read alignment. I'm not suggesting a re-analysis of these data with a broader in silico reference sequence collection, but I think the discussion should address this possible bias, i.e. false-negative results for divergent ARGs because of the algorithm/reference choice.
This comment of reviewer 1 is similar to the second comment of reviewer 3, where we have added a discussion of how we could compare and add more recent metagenomic samples to our work. See lines 311-313 in the manuscript.

As mentioned above, the manuscript has little in the assessment of ARG transmission patterns, which is fine as it was not the major point of the paper, but Zhang et al. (PMID 34362925; Nature Communications 12: 4765) & Zhang et al. (PMID 35322038; Nature Communications 13: 1553) have recently published some large scale ARG metagenomic analyses that included assessment of ARG transmission and generation of risk metrics. At a minimum, the discussion should place the author's work in the context of these recent efforts.

We were not familiar with these two examples of work but found them very interesting. Therefore, we have added the two mentioned references in the context of comparing our to other types of data, see lines 272-274.

As mentioned above, the data include ARGs that have as little as a single read fragment aligned. The authors should add a statement that they are including these data for complete transparency so others can decide their own cut-offs when analyzing the data.

We have added this in our discussion of things to keep in mind when using the data resource in lines 284-287.

No information is given in the discussion on the long-term maintenance of this resource. What is the plan as new metagenomics data become available? CARD has (beta) pathogen-of-origin kmer tools for ARGs, will the authors be exploring similar methods to provide a more pathogen-centric perspective in future analyses?
These are good points to highlight in our discussion, so we have added these in lines 289-294, where we mention that there are even more metagenomic samples to add and other approaches to compare with.

MINOR POINTS

Figure 1C caption should mention the number of samples for which the collection date was NULL.
We have added this information for sampling location and collection date in the legend of figure 1.

Lines 210-214 have very confusing grammar.
We have rewritten these paragraphs' lines to be more precise; see lines 217-220.

The phrase "ARG template" is used without proper definition.
We have changed our use of "ARG template" to instead "ARG reference sequences" and other similar variations in the manuscript.

Reply to reviewer 2
The resource presented by Martiny et al. is timely and has potential to boost the research antimicrobial resistance through widening access. The methods are presented clearly, and the datasets are made publicly available. There are a few points that I recommend the authors to consider towards improving the quality through cross-checking some of the analyses.

1. Given the impressive volume and the breadth of the data analysed, it is quite surprising that 96 ARGs did not have any alignments. A cross-cheling of these results and any indicators of the underlying reasons (e.g., these ARGS being very specific to the environments not being represented here?) will be important.
   The ARGs that did not have any matches are all closely related variants to an ARG that were detected, so we were not surprised that none of the read fragments aligned to these.

2. Figure 4c, what do the rows 'Metagenome' and 'Metagenomes' refer to? Some error in metadata curation?
   Good question, although we do not have any answer to what these two labels refer to. They are simply what the data has been annotated as, and we chose to include these two labels in the figure to be as transparent as possible. It could be argued that these should be merged or ignored, but we leave that up to the user.

3. Fosfomycin ARG (green in Figure 4C), seems quite high in Food Metagenomes, while it is barely present in panels A and B. I suppose this indicates uneven distribution of sampling 'host'? Also, is there known connection between food microbiomes and

fosfomycin resistance? BTW, 'environment' will be a much better and accurate term than 'host'.
This is correct, as most of the metagenomes annotated as food metagenomes are either missing location and sampling date labels or that they belong to some of the largest groups. Regarding the comment on using 'environment' as the category, we have chosen to use the host as the overall category in the shared data tables, but in the manuscript, refer to the sampling origin as host or environment.

Minor comments:

1. Line 81: uploaded between 2010-01-01 and 2020-01-01 that had library source as 'METAGEOMIC'
Thanks, this has been corrected.

2. Inconsistent line spacing starting on page 5 line 135 through page 6 line 152
The line spacing has been corrected for the corresponding lines.

3. Any data on how sequencing depth affects detection of ARGs or 16/18S genes? For example, Fig2a could be converted from a density plot to a scatterplot showing sequencing depth vs fragments
Unfortunately, the sequencing depth is not recorded in the metadata by ENA, so we cannot accommodate this request, despite it being an excellent point. Instead, we have now added a new supplementary figure (Fig S3) that shows the number of ARG/rRNA fragments aligned compared to the raw base count (lines 184-185).

4. Figure 3, what do different colour shades mean for boxes?
The shades do not mean anything, only to distinguish that the boxes are for different categorical values.

<u>Reply to reviewer 3</u>

My opinion on that paper is that it's a valuable analysis, and seems to have been done carefully. I had only two technical quibbles:

1. The manuscript does not explain how the analysis workflow handles two key issues. First: AMR databases are full of different versions of the same gene - e.g. there are more than 170 allelic versions of the CTX-M gene. Were all reads mapped to a database containing all of these, or were representatives chosen? If mapping to everything, what was done with reads that mapped to multiple alleles of one gene , and how were counts resolved? Two: I don't understand why, when calculating abundance, using counts of reads mapping to ribosomal RNA as a denominator makes sense, as rRNA arrays are different lengths in different species.

   All the read fragments were mapped to all ARG sequences in the ResFinder database, meaning that we did indeed map to multiple versions of the same gene. KMA was specifically created to resolve this issue of redundancy by using a specific sorting scheme that can distinguish between homologous sequences and choose the most parsimonious templates.

Regarding the second point, we chose to use the rRNA counts as the denominator due to working with acquired antimicrobial resistance genes. Since they are acquired by the bacteria, and we want to know how much ARGs the bacteria carry, we decided to compare the ratio of ARG counts to rRNA counts. Another common denominator option is the total number of fragments, but since the bacterial contribution can vary drastically across environments (relative to e.g. eukaryotes, viruses and archaea), that would not be appropriate.

2. The text seems to suggest the same mapping workflow was used for nanopore, pacbio, and illumina. Is this really true? The same kmer size also? If yes, a lot of sensitivity will have been lost in the long read data , although since this is <10% of the data, this is not really a big issue.

   This is true, as we chose to use the same workflow regardless of which instrument the sequencing reads were generated on. But the reviewer raises a good point, which we have now added in the discussion; see lines 294-296.

I also had one red flag: Given the high rate of metadata errors in the ENA, I am suspicious of the samples dated between 1845 and 1905 in Figure 3 - is there a way to check these? If there is no associated publication discussing old metagenomes, I would honestly consider discarding those datapoints as mislabelled.
We have not yet come up with a way to control sampling dates beside a manual curation, but it is good to question whether the metadata is correct or mislabelled. We have added this point in the discussion in lines 279-281.

Figure 4 is great, v interesting!

Thank you. We agree!
